# Establishment of epidemiological cutoff values against *Fonsecaea monophora*, an agent of chromoblastomycosis and cerebral phaeohyphomycosis

**DOI:** 10.1128/jcm.01846-25

**Published:** 2026-06-25

**Authors:** Dallas J. Smith, Tijmen van Beurden, Yalong Li, Rodrigo Almeida-Paes, Bruna Jacomel, Vania Aparecida Vicente, Bruno P. R. Lustosa, Thiago Henrique Walter, Elias Nunes Monteiro Neto, Vinaykumar Hallur, Diptanu Paul, Nimmy Paul, Ruoyu Li, Sha Lu, Sarah E. Kidd, Alexandro Bonifaz, Arghadip Samaddar, Rogelio de J. Treviño-Rangel, Gloria M. González, Anuradha Chowdhary, José Guillermo Pereira Brunellie, Guillermo García-Effron, Shivaprakash M. Rudramurthy, Harsimran Kaur, Arunaloke Chakrabarti, Conceicão de Maria Pedrozo e Silva de Azevedo, Sirlei Garcia Marques, Daniel Wagner C. L. Santos, Regielly C. R. Cognialli, Flávio Queiroz-Telles, Bram Spruijtenburg, Eelco F. J. Meijer, Theun de Groot, Amir Seyedmousavi, Philippe J. Dufresne, Shawn R. Lockhart, Nathan P. Wiederhold, Yinggai Song, Ferry Hagen

**Affiliations:** 1 Mycotic Diseases Branch, Centers for Disease Control and Prevention 1242, Atlanta, Georgia, USA; 2 Westerdijk Fungal Biodiversity Institute, Utrecht, The Netherlands; 3 Department of Dermatology and Venerology, Peking University First Hospital 26447, Beijing, China; 4 Research Center for Medical Mycology, Peking University 12465, Beijing, China; 5 National Clinical Research Center for Skin and Sexually Transmitted Diseases 599175, Beijing, China; 6 Beijing Key Laboratory of Molecular Diagnosis on Dermatoses, Beijing, China; 7 Laboratório de Micologia, Instituto Nacional de Infectologia Evandro Chagas, Fundação Oswaldo Cruz, Fiocruz 735822 , Rio de Janeiro, Brazil; 8 Postgraduate Program of Microbiology, Parasitology and Pathology, Federal University of Parana 28122, Curitiba, Brazil; 9 Radboudumc-CWZ Center of Expertise for Mycology 6034, Nijmegen, the Netherlands; 10 Post-graduation Program of Engineering Bioprocess and Biotechnology, Federal University of Parana 28122, Curitiba, Brazil; 11 Department of Microbiology, All India Institute of Medical Sciences 410775, Bhubaneswar, Odisha, India; 12 Department of Microbiology, Government Medical College 29319, Kottayam, Kerala, India; 13 Department of Dermatology, Sun Yat-Sen Memorial Hospital, Sun Yat-Sen University 548099, Guangzhou, China; 14 National Mycology Reference Centre, SA Pathology 10107, Adelaide, Australia; 15 School of Biological Sciences, Adelaide University 695511, Adelaide, Australia; 16 Servicio de Dermatología, Hospital General de México “Dr. Eduardo Liceaga” 61575, Mexico City, Mexico; 17 Department of Neuromicrobiology, National Institute of Mental Health and Neurosciences 489654, Bengaluru, Karnataka, India; 18 Departamento de Microbiología, Facultad de Medicina, Universidad Autónoma de Nuevo León 648569, Monterrey, Nuevo Leon, Mexico; 19 Medical Mycology Unit, Department of Microbiology, Vallabhbhai Patel Chest Institute, University of Delhi 90935, Delhi, India; 20 National Reference Laboratory for Antimicrobial Resistance in Fungal Pathogens, Vallabhbhai Patel Chest Institute, University of Delhi 90935, Delhi, India; 21 Center for Dermatological Diseases, Ministry of Public Health and Social Welfare, San Lorenzo, Paraguay; 22 Laboratorio de Micología y Diagnóstico Molecular, Cátedra de Parasitología y Micología, Facultad de Bioquímica y Ciencias Biológicas, Universidad Nacional del Litoral 28247, Santa Fe, Argentina; 23 Consejo Nacional de Investigaciones Científicas y Tecnológicas, CCT Santa Fe 199757 , Santa Fe, Argentina; 24 Department of Medical Microbiology, Postgraduate Institute of Medical Education and Research: Chandigarh 685007 , Chandigarh, India; 25 Doodhadhari Burfani Hospital and Research Institute, Haridwar, Uttarakhand, India; 26 Post-graduation Program of Health Science, Federal University of Maranhão 37892, São Luís, Maranhão, Brazil; 27 Department of Infectious Diseases and Infection Control, Federal University of Maranhão, EBSERH 37892, São Luís, Maranhão, Brazil; 28 Laboratório Cedro, São Luís, Maranhão, Brazil; 29 Instituto D´Or de Pesquisa e Ensino, IDOR 519983 , Rio de Janeiro, Brazil; 30 Hospital de Clínicas, Federal University of Paraná 28122, Curitiba, Brazil; 31 Department of Public Health, Federal University of Paraná 28122, Curitiba, Brazil; 32 Department of Medical Microbiology and Immunology, Canisius-Wilhelmina Hospital (CWZ)/Dicoon 6030 , Nijmegen, the Netherlands; 33 Microbiology Service, National Institutes of Health Clinical Center 24481, Bethesda, Maryland, USA; 34 Laboratoire de santé publique du Québec, Institut National de Santé Publique du Québec, Sainte-Anne-de-Bellevue 54470 , Québec, Canada; 35 Department of Pathology and Laboratory Medicine, The University of Texas Health Science Center 12345, San Antonio, Texas, USA; 36 Department of Medical Microbiology, University Medical Center Utrecht 647220, Utrecht, the Netherlands; University of Calgary, Calgary, Alberta, Canada

**Keywords:** antifungal resistance, antifungal susceptibility testing, epidemiological cutoff value, *Fonsecaea*

## Abstract

*Fonsecaea monophora* is a dematiaceous fungus with worldwide distribution that can cause chromoblastomycosis, an implantation mycosis and a neglected tropical disease. *F. monophora* also causes phaeohyphomycosis, including central nervous system infections like brain abscesses, with high mortality rates. In the absence of clinical breakpoints, epidemiological cutoff values (ECVs) can aid clinicians in monitoring antifungal resistance trends and guide initial antifungal selection. To establish *F. monophora* MIC distribution and ECVs, we performed antifungal susceptibility testing (AFST) on *F. monophora* isolates. AFST data on *F. monophora* isolates were collected at 14 laboratories during September 2023–May 2026. Species identification was previously confirmed by DNA sequence analysis. AFST was performed by CLSI M38 standard broth microdilution method for itraconazole, voriconazole, posaconazole, isavuconazole, ketoconazole, terbinafine, flucytosine, and amphotericin B. The ECVs were established using the iterative statistical method with ECOFFinder (v2.1) following CLSI M57 guidelines. We analyzed MIC results from 137 *F. monophora* isolates. The calculated ECVs were itraconazole, 2 μg/mL; voriconazole, 0.25 μg/mL; posaconazole, 1 μg/mL; isavuconazole, 0.25 μg/mL; and terbinafine, 0.5 μg/mL. Amphotericin B and flucytosine had bimodal distributions, and no ECVs were set. Ketoconazole did not have data from ≥100 isolates. These *F. monophora* ECVs can detect non-wild-type isolates to reevaluate antifungal treatment and investigate treatment failure associated with potential clinical resistance. Established through a global multicenter collaboration, these values provide a baseline to better understand the *in vitro* antifungal susceptibility profile of this species and monitor resistance.

**IMPORTANCE:**

*Fonsecaea monophora* is a dematiaceous fungus with worldwide distribution that can cause chromoblastomycosis, an implantation mycosis and neglected tropical disease of the skin and subcutaneous tissue. *F. monophora* also causes phaeohyphomycosis, including central nervous system infections like brain abscesses with high mortality rates. The paucity of antifungal susceptibility testing data on *F. monophora* complicates interpretation of minimum inhibitory concentration (MIC) values. We performed antifungal susceptibility testing on 137 *F. monophora* isolates to establish MIC distributions and epidemiological cutoff values (ECVs) for eight antifungals, including those commonly used to treat *F. monophora* infections. The calculated ECVs for commonly used antifungals were 2 μg/mL for itraconazole, 0.25 μg/mL for voriconazole, 1 μg/mL for posaconazole, and 0.5 μg/mL for terbinafine. ECVs can assist in selecting potential treatments for *F. monophora* and tracking antifungal resistance trends.

## INTRODUCTION

*Fonsecaea monophora* is a dematiaceous fungus with worldwide distribution that can cause chromoblastomycosis, an implantation mycosis of the skin and subcutaneous tissue and a World Health Organization-recognized neglected tropical disease (1, 2). *F. monophora* also causes phaeohyphomycosis, including central nervous system (CNS) infections, such as brain abscesses, that have high mortality rates (3, 4). Morphological identification only enables the identification of *Fonsecaea* to the genus level; molecular sequencing of the internal transcribed spacer (ITS) or D1/D2 region of the ribosomal DNA is needed to distinguish *F. monophora* from other closely related species (e.g., *Fonsecaea pedrosoi* and *Fonsecaea nubica*) (5). Extensive environmental sampling to determine ecological niches of *F. monophora* is lacking, but limited environmental screening using molecular detection has found *F. monophora* on various plants, including babassu coconut (6).

Chromoblastomycosis treatment guidelines are not available, but months to years of antifungal treatment, coupled with surgery, are often required. In general, itraconazole is commonly considered first-line therapy for chromoblastomycosis, but the optimal therapy for infections caused by *F. monophora* is unknown (7–10) and limited to case reports. Terbinafine, in combination with itraconazole, and voriconazole have been used successfully in treating chromoblastomycosis caused by *F. monophora* (11, 12). Non-pharmacological treatment (e.g., photodynamic therapy), in combination with antifungals, has been used successfully in refractory disease (13), although this therapy is not widely available. The European Fungal Infection Study Group of the European Society of Clinical Microbiology and Infectious Diseases has developed guidelines for phaeohyphomycosis and recommends voriconazole (preferred due to brain tissue penetration) or posaconazole for cerebral abscesses, regardless of causative fungus, coupled with surgical intervention (4), but these antifungals are often not available in resource-limited settings.

Antifungal minimum inhibitory concentration (MIC) data for *F. monophora* are limited. Previous reports were from a small number of isolates from single centers using different antifungal susceptibility testing (AFST) methodologies; results indicated low MICs against mold-active triazoles but trended higher for itraconazole (11, 14, 15). While data are limited, MICs for *F. monophora* causing CNS infections seem to be similar to isolates causing chromoblastomycosis (16–20).

Epidemiological cutoff values (ECVs) may be used to predict if a fungal isolate is non-wild type in regard to its *in vitro* response to an antifungal (21). ECVs can be a useful tool to monitor for antifungal resistance, especially in patients with chromoblastomycosis caused by *F. monophora*, which may lead to years of antifungal treatment (22). They are strictly based on *in vitro* MIC distribution with no clinical outcome or pharmacokinetic and pharmacodynamic data. They are not intended to provide a replacement for breakpoints but may assist clinicians in making more rational decisions, as non-wild-type strains may not respond to a specific antifungal therapy the same way as a wild-type strain of the same species. To date, the only dematiaceous fungi with Clinical Laboratory Standards Institute (CLSI) ECVs are *F. pedrosoi*, the most commonly reported agent of chromoblastomycosis (23). Thus, we performed AFST on *F. monophora* isolates to establish their MIC distributions, ECVs, percentage non-wild type, and modal MICs for eight antifungals: itraconazole, voriconazole, posaconazole, isavuconazole, ketoconazole, terbinafine, flucytosine, and amphotericin B.

## RESULTS

AFST was performed by broth microdilution (BMD), as outlined in the CLSI reference standard M38 Ed3 for filamentous fungi (24). One hundred and thirty-seven *F. monophora* isolates originating from 14 different countries were tested in 14 different laboratories (Table 1). ECVs were determined using the iterative statistical method with ECOFFinder (v2.1) following CLSI M57 guidelines (21, 25). The calculated ECVs were: 2 μg/mL for itraconazole, 0.25 μg/mL for voriconazole, 1 μg/mL for posaconazole, 0.25 μg/mL for isavuconazole; and 0.5 μg/mL for terbinafine (Table 2). Amphotericin B and flucytosine had bimodal MIC distributions, therefore, no ECVs were set. Ketoconazole did not have data from ≥100 isolates to establish an ECV.

**TABLE 1:** Minimum inhibitory concentrations for *Fonsecaea monophora* and eight antifungals

Antifungal (no. of isolates tested at nine labs)^*a*^	No. of isolates per MIC (μg/mL)											
	≤0.016	0.03	0.06	0.12	0.25	0.5	1	2	4	8	16	≥32
Itraconazole (131)		8	5	10	44	**45**	15	3			1	
Voriconazole (137)		24	**52**	37	16	4		2	1		1	
Posaconazole (126)	3	15	22	20	**39**	21	3	1			2	
Isavuconazole (106)	1	14	**36**	28	10	8	5		2	1	1	
Ketoconazole (62)			10	21	**24**	4	2			1		
Terbinafine (117)	17	14	24	**39**	16	3	2	2				
Flucytosine (118)					1	7	15	**26**	**26**	23	6	14
Amphotericin B (137)			1	1	3	6	22	31	16	**51**	6	

^*a*^ Some laboratories were unable to perform antifungal susceptibility testing on all antifungals due to inability to test an antifungal or difficulties with an *F. monophora* isolate. Modal MICs are in bold.

**TABLE 2:** Epidemiological cutoff values and minimum inhibitory concentration characteristics for *Fonsecaea monophora* and eight antifungals^*c*^

	ECV (μg/mL)	NWT (%)	Modal MIC (μg/mL)
Itraconazole	2	1	0.5
Voriconazole	0.25	6	0.06
Posaconazole	1	2	0.25
Isavuconazole	0.25	16	0.06
Ketoconazole	Not set	N/A^*b*^	0.12
Terbinafine	0.5	3	0.12
Flucytosine	Not set^*a*^	N/A^*b*^	2, 4
Amphotericin B	Not set^*a*^	N/A^*b*^	8

^*a*^ Amphotericin B and flucytosine showed bimodal distribution and strong inter-lab variation for *Fonsecaea monophora*. A reliable epidemiological cutoff value could not be set.

^*b*^ Not applicable because no epidemiological cutoff value was established.

^*c*^ ECV, epidemiological cutoff value; NWT, non-wild type; MIC, minimum inhibitory concentration; N/A, not applicable.

The percentage of non-wild-type isolates (MICs greater than the established ECV) was 1% (1/131) for itraconazole, 6% (8/137) for voriconazole, 2% (3/124) for posaconazole, 16% (18/106) for isavuconazole, and 3% (4/117) for terbinafine (Table 2). Modal MICs were 0.5 μg/mL for itraconazole, 0.06 μg/mL for voriconazole and isavuconazole, 0.25 μg/mL for posaconazole, 0.12 μg/mL for ketoconazole and terbinafine, 2 and 4 μg/mL for flucytosine, and 8 μg/mL for amphotericin B. The MIC distributions were 0.03–16 μg/mL for itraconazole and voriconazole, 0.016–16 μg/mL for posaconazole and isavuconazole, 0.06–8 μg/mL for ketoconazole, 0.008–2 μg/mL for terbinafine, 0.25 to ≥32 µg/mL for flucytosine, and 0.06–16 μg/mL for amphotericin B. Sequential isolates, not included in ECV calculation, for three unique patients showed one- to twofold MIC dilution differences over time for seven antifungals (Table S1).

## DISCUSSION

Through a global network of laboratories, we established ECVs for *F. monophora* according to CLSI BMD methodology for commonly used and guideline-recommended antifungals for both chromoblastomycosis and cerebral phaeohyphomycosis. Data from ≥100 isolates were collected from six continents and were tested in 14 independent laboratories, strengthening the applicability of these results. Treatment for infections caused by *F. monophora* is challenging; these ECVs are a tool for selecting appropriate antifungal therapy, in addition to pharmacokinetics and pharmacodynamics, site of infection, lesion size, and patients’ health status (e.g., Dectin-1 deficiency and CARD9 mutations) (8, 26–28). In the absence of breakpoints, these ECVs can also be used to monitor changes in susceptibility.

Randomized controlled trials for chromoblastomycosis have not been conducted, and first-line therapy has largely depended on open and non-comparative clinical trials and case reports. Differences in clinical response rates to antifungals by various causative organisms in the same genus have not been evaluated. Our data suggest that *F. monophora* may respond differently *in vitro* to certain commonly used antifungals than *F. pedrosoi*, the most commonly reported cause of chromoblastomycosis globally (29). In particular, the ECV (2 μg/mL vs 0.5 μg/mL) and modal MIC (0.5 μg/mL vs 0.12 μg/mL) for itraconazole against *F. monophora* are twofold dilutions higher than against *F. pedrosoi* (23). Isavuconazole, uncommonly used for chromoblastomycosis, had a twofold lower ECV for *F. monophora* compared to *F. pedrosoi* (0.25 μg/mL vs 1 μg/mL). For other antifungals, the ECVs were similar between *F. monophora* and *F. pedrosoi*. Antifungals like isavuconazole or terbinafine may be more appropriate first-line treatment options for chromoblastomycosis caused by *F. monophora* based on these laboratory findings, but clinical data are needed to correlate *in vivo* and *in vitro* results. Furthermore, chromoblastomycosis often occurs in low- and middle-income countries where these newer azoles or terbinafine is neither affordable nor available (30). Finally, molecular sequencing is critical to differentiate between *F. monophora* and *F. pedrosoi*, which is often unavailable in countries where these dematiaceous fungi are more common (5). Identification at the *Fonsecaea* genus level would not allow these ECVs to be used appropriately, as we note substantial differences in MIC profiles between the two species (23).

Clinical guidelines for systemic phaeohyphomycosis causing cerebral abscesses, regardless of causative fungus, marginally recommend voriconazole or posaconazole but recommend against the use of amphotericin B (4). The modal MIC or ECV established in our study for *F. monophora* against these azoles and amphotericin B supports these guideline recommendations. A murine model also demonstrated that mice infected with *F. monophora* had better clearance of the fungus in brain tissue with posaconazole compared with amphotericin B and itraconazole (31).

Our results demonstrated a low prevalence (1%–6%) of non-wild-type isolates for itraconazole, voriconazole, posaconazole, and terbinafine, suggesting a low propensity for *F. monophora* to acquire molecular mechanisms of resistance, although isavuconazole had a high prevalence of non-wild-type isolates at 16%. No microbiological evidence of antifungal resistance has been reported for *F. monophora*, although cases refractory to antifungal treatment have been published (13, 32). In our study, one isolate had MICs of 16 μg/mL for itraconazole, voriconazole, posaconazole, and isavuconazole, suggesting microbiological resistance, but clinical data were not available, and resistance mutations were not investigated. The ECVs and modal MICs established here can assist in monitoring MICs associated with an infection over time to identify when increased MICs become non-wild type, provide clues to treatment pressure leading to treatment failure, or serve as early warning indicators of isolates with genetic changes from environmental acquisition of the fungus. Increasing MICs in sequential *F. monophora* isolates with antifungal exposure have not been reported. However, one study found two patients with *F. pedrosoi* infections in which clinical resistance and treatment failure occurred after sequential isolates showed MICs increasing substantially (33). In our study, three unique patients had sequential *F. monophora* isolates tested, and only one- or twofold dilution differences (increases and decreases) in MICs over time for multiple antifungals were observed, although no clinical data were available to evaluate antifungal use and length of therapy. Whole genome sequencing of non-wild-type isolates could potentially identify genetic mutations leading to higher MICs when compared to wild-type isolates (34).

The interpretation of these data and ECVs is subject to at least six limitations. First, the characteristic thick-walled, single, or multicellular clusters of pigmented fungal cells (also known as medlar bodies, muriform cells, or sclerotic bodies) seen in chromoblastomycosis caused by *F. monophora* were not used as the inoculum and may respond differently to exposure to antifungals than hyphae and conidia (7). Second, concentrations of certain antifungals (e.g., itraconazole and terbinafine) in skin tissue and appendages may be higher than that in serum. Thus, higher concentrations at the site of these skin infections may be achievable for some drugs. Third, longer incubation periods were needed (4–6 days) for *F. monophora* in our study than in established CLSI protocols. Fourth, while this is the largest AFST study of *F. monophora*, it still represents a convenience sample and may not represent the susceptibility profile of isolates globally. Fifth, clinical information were not available for most isolates, and we were unable to investigate potential differences between isolates from patients on prolonged antifungal treatment or isolates that caused cerebral disease versus chromoblastomycosis; however, limited data suggest susceptibility profiles are comparable (16–20). Sixth, CLSI requires 100 isolates to be tested per antifungal to establish an ECV (21); we were not able to set an ECV for ketoconazole.

### Conclusion

*F. monophora* can cause disfiguring chromoblastomycosis and cerebral phaeohyphomycosis with high mortality rates. ECVs were established for *F. monophora* for common antifungals used in treatment including itraconazole (ECV: 2 μg/mL),voriconazole (ECV: 0.25 μg/mL), posaconazole (ECV: 1 μg/mL), and terbinafine (ECV: 0.5 μg/mL) and can assist in selecting potential treatment options for this fungus. More research is needed to understand susceptibility differences within the *Fonsecaea* genus and further awareness of using these ECVs in clinical laboratories globally.

## MATERIALS AND METHODS

### Isolates

Species identification was previously confirmed by DNA analysis using ITS or ITS and D1/D2 sequencing. Among the 137 *F. monophora* isolates, 52 came from China, 34 from Brazil, 19 from the United States and Puerto Rico, 10 from India, 5 from Mexico, 3 each from Australia and Cuba, 2 each from Canada and the Netherlands, and 1 each from France, Paraguay, Spain, South Africa, United Kingdom. Two isolates were from South America without a specified country. The countries in which the isolates were tested do not necessarily represent the countries where the infections were acquired.

Isolate data were submitted by 14 different laboratories on four continents: 43 isolates submitted by the Research Center for Medical Mycology, Peking University, Beijing, China; 34 from the Westerdijk Fungal Biodiversity Institute, Utrecht, The Netherlands; 17 from the Microbiological Collections of Paraná Network (CMRP-Taxonline), Federal University of Paraná, Curitiba, Brazil; 11 from the Fungus Testing Laboratory, University of Texas Health Science Center, San Antonio, TX, USA; 10 from the Laboratório de Micologia, Instituto Nacional de Infectologia Evandro Chagas, Fundação Oswaldo Cruz, Fiocruz, Rio de Janeiro, RJ, Brazil; 4 from the Department of Laboratory Medicine, National Institute of Health, Bethesda, MD, USA; 4 from Departamento de Microbiología, Facultad de Medicina, Universidad Autónoma de Nuevo León, Monterrey, Nuevo León, Mexico; 4 from the Department of Medical Microbiology, Postgraduate Institute of Medical Education and Research, Chandigarh, India; 3 from the National Reference Laboratory for Antimicrobial Resistance in Fungal Pathogens, Vallabhbhai Patel Chest Institute, University of Delhi, Delhi, India; 2 from Laboratoire de Santé Publique du Québec, Québec, Canada; 2 from Department of Neuromicrobiology, National Institute of Mental Health and Neuro Sciences, Bengaluru, Karnataka, India; 1 from ARUP Laboratories, Salt Lake City, UT, USA; 1 from Servicio de Dermatología, Hospital General de México “Dr. Eduardo Liceaga,” Mexico City, Mexico; and 1 from Laboratorio de Micología y Diagnóstico Molecular, Cátedra de Parasitología y Micología, Facultad de Bioquímica y Ciencias Biológicas, Universidad Nacional del Litoral, Santa Fe, Argentina.

### Antifungal susceptibility testing

One-hundred and thirty-seven *F. monophora* isolates were used in this study. AFST was performed by broth microdilution as outlined in the CLSI reference standard M38 Ed3 for filamentous fungi (24). Isolates used as reference strains or quality controls were *Aspergillus fumigatus* ATCC MYA 3626, *Aspergillus flavus* ATCC 204304, *Candida parapsilosis* ATCC 22019 (=CBS 604), *Pichia kudriavzevii* ATCC 6258 (=CBS 573), *Hamigera insecticola* ATCC MYA-3630, and *Paecilomyces variotii* ATCC MYA-3630; quality controls were within M38M51S CLSI ranges. Itraconazole, voriconazole, posaconazole, isavuconazole, ketoconazole, terbinafine, flucytosine, and amphotericin B were tested. The MICs were determined visually after 48–144 hours (2–6 days, depending on the growth rate of the individual isolate) of incubation at 35°C. The inoculum was adjusted with an absorbance of 530 nm at 0.15–0.17 and verified by hematocytometer counting to be within 0.2–2.5 × 10^6^ conidia/mL or by Cellometer X2 (Nexcelom, Manchester, United Kingdom). This inoculum was then used for further processing. The antifungal susceptibility endpoint for itraconazole, voriconazole, posaconazole, isavuconazole, ketoconazole, terbinafine, and amphotericin B was 100% inhibition of growth. For flucytosine, ≥50% inhibition of growth compared to the positive control growth was used.

### Epidemiological cutoff values

The ECVs were established using the iterative statistical method with ECOFFinder (v2.1) with a 97.5% threshold following CLSI M57 guidelines (21, 25). Percentage non-wild type, and modal MIC were calculated for each antifungal.

## SUPPLEMENTAL MATERIAL

Table S1 jcm.01846-25-s0001.docx Minimum inhibitory concentration characteristics for *Fonsecaea monophora* sequential isolates from three unique patients and seven antifungals.

ASM does not own the copyrights to Supplemental Material that may be linked to, or accessed through, an article. The authors have granted ASM a non-exclusive, world-wide license to publish the Supplemental Material files. Please contact the corresponding author directly for reuse.
