# Supplementary material for: Establishment of epidemiological cutoff values against Fonsecaea monophora, an agent of chromoblastomycosis and cerebral phaeohyphomycosis
Source: J Clin Microbiol. 2026 Jun 25;64(8):e01846-25. doi: 10.1128/jcm.01846-25 (PMC13464324; doi:10.1128/jcm.01846-25)
Supplement: Supplementary file 1 [file jcm.01846-25-s0001.docx]

**Supplemental Table 1: Minimum inhibitory concentration characteristics for *Fonsecaea monophora* sequential isolates from three unique patients and seven antifungals***

|  | Itraconazole (μg/mL) | Voriconazole (μg/mL) | Posaconazole (μg/mL) | Ketoconazole (μg/mL) | Terbinafine (μg/mL) | Flucytosine (μg/mL) | Amphotericin B (μg/mL) |
| --- | --- | --- | --- | --- | --- | --- | --- |
| **Patient #1** |  | | | | | | |
| Initial isolate | 0.25 | 0.25 | 0.25 | 0.25 | 0.12 | 32 | 2 |
| 2^nd^ isolate | 0.5 | 0.12 | 0.25 | 0.25 | 0.12 | >64 | 8 |
| 3^rd^ isolate | 0.5 | 0.25 | 0.25 | 0.25 | 0.03 | 8 | 2 |
| 4^th^ isolate | 0.25 | 0.25 | 0.25 | 0.25 | 0.25 | >64 | 8 |
| 5^th^ isolate | 0.25 | 0.25 | 0.5 | 0.5 | 0.25 | 32 | >16 |
| 6^th^ isolate | 0.25 | 0.25 | 0.25 | 0.25 | 0.12 | 32 | 4 |
| 7^th^ isolate | 0.5 | 0.25 | 0.25 | 0.25 | 0.12 | 32 | 4 |
| 8^th^ isolate | 0.5 | 0.25 | 0.25 | 0.25 | 0.12 | 32 | 8 |
| 9^th^ isolate | 0.25 | 0.25 | 0.25 | 0.25 | 0.12 | >64 | 8 |
| 10^th^ isolate | 0.25 | 0.25 | 0.5 | 0.25 | 0.25 | >64 | 8 |
| 11^th^ isolate | 0.5 | 0.25 | 0.5 | - | 0.12 | 32 | 8 |
| 12^th^ isolate | 0.25 | 0.25 | 1 | 0.5 | 0.25 | 32 | 8 |
| 13^th^ isolate | 0.25 | 0.25 | 0.5 | 0.25 | 0.25 | >64 | 4 |
| 14^th^ isolate | 0.25 | 0.25 | 0.25 | 0.25 | 0.25 | >64 | 8 |
| **Patient #2** |  | | | | | | |
| Initial isolate | 0.5 | 0.25 | 0.25 | 0.25 | 0.25 | 16 | 8 |
| 2^nd^ isolate | 0.25 | 0.25 | 0.25 | 0.25 | 0.12 | 8 | 2 |
| **Patient #3** |  |  |  |  |  |  |  |
| Initial isolate | 0.25 | 0.25 | 0.5 | 0.25 | 0.25 | >64 | 8 |
| 2^nd^ isolate | 0.5 | 0.12 | 0.12 | 0.25 | 0.25 | 32 | 8 |
| 3^rd^ isolate | 0.25 | 0.12 | 0.12 | 0.25 | 0.25 | 32 | 8 |

*Sequential isolate data were obtained from a single laboratory in Brazil. Clinical and epidemiological data were not available. Only initial isolate’s data were included in epidemiological cutoff value calculation.
